# Different Effects of Myoinositol plus Folic Acid versus Combined Oral Treatment on Androgen Levels in PCOS Women

**DOI:** 10.1155/2016/3206872

**Published:** 2016-11-02

**Authors:** Ali Cenk Ozay, Ozlen Emekci Ozay, Recep Emre Okyay, Erkan Cagliyan, Tuncay Kume, Bulent Gulekli

**Affiliations:** ^1^Aksehir State Hospital, Konya, Turkey; ^2^Department of Obstetrics & Gynecology, Medical School, Dokuz Eylul University, Izmir, Turkey; ^3^Department of Biochemistry, Medical School, Dokuz Eylul University, Izmir, Turkey

## Abstract

Recently, myoinositol (myo-ins) and folic acid combination has gained an important role for treating Polycystic Ovary Syndrome (PCOS), in addition to combined oral contraceptives (COC). We aimed to examine myo-ins effects on anti-Mullerian hormone (AMH) levels and compare them with those ones obtained administering COC. In this prospective study, 137 PCOS patients, diagnosed according to Rotterdam criteria and admitted to the Reproductive Endocrinology and Infertility Outpatient Clinic at Dokuz Eylul University (Izmir, Turkey), were included. After randomization to COC (*n* = 60) and myo-ins (*n* = 77) arms, anthropometric measurements, blood pressure, Modified Ferriman Gallwey scores were calculated. Biochemical and hormonal analysis were performed, and LH/FSH and Apo B/A1 ratios were calculated. Data analysis was carried out in demographically and clinically matched 106 patients (COC = 54; myo-ins = 52). After 3-month treatment, increase in HDL and decreases in LH and LH/FSH ratio were statistically more significant only in COC group when compared with baseline (in both cases *p* > 0.05). In myo-ins group, fasting glucose, LDL, DHEAS, total cholesterol, and prolactin levels decreased significantly (for all *p* < 0.05). Progesterone and AMH levels, ovarian volume, ovarian antral follicle, and total antral follicle counts lessened significantly in both groups (for all *p* < 0.05). In PCOS treatment, MYO is observed more effective in reductions of total ovarian volume and AMH levels.

## 1. Introduction

Anti-Mullerian hormone (AMH), a polypeptide, secreted by the granulosa cells of the preantral and early developing antral follicles, has been shown to be a predictor of ovarian activity [1]. PCOS patients show an increased number of antral follicles; therefore they have higher circulating AMH levels than the healthy women [2]. The interactions between AMH and the hormonal profile characteristics in PCOS require a better assessment. The positive correlation between insulin resistance and serum AMH levels suggests that insulin exerts an action on AMH synthesis; however this aspect is not yet fully understood [3].

Polycystic Ovary Syndrome (PCOS) is a common endocrine disorder, affecting 6–10% of women in reproductive age. This syndrome is characterized by biochemical or clinical signs of hyperandrogenism, chronic anovulation, and polycystic ovaries [4]. It is frequently associated with insulin resistance and obesity. Evidence suggests that insulin resistance and its compensatory hyperinsulinemia play an important role in PCOS pathogenesis [5, 6]. Insulin is associated with hyperandrogenism; it acts synergistically with luteinizing hormone to increase the androgen production of theca cells [7]. Therefore, administration of insulin sensitizers ameliorates hyperandrogenemia and ovulatory functions [8].

Inositol (hexahydroxycyclohexane) belongs to the vitamin B complex group; it is a 6-carbon ring compound, having a hydroxyl group linked to each carbon of the ring, with nine possible stereoisomeric forms depending on the epimerization of the six hydroxyl groups. Among them, myoinositol (myo-ins) is the mostly represented isoform, with very relevant biological functions [9]. Increasing evidence has demonstrated that myo-ins plays a key role in cell morphogenesis and cytogenesis, lipid synthesis, structure of cell membranes, and cell growth [10]. Myo-Ins administration improves hormonal profile, oocyte maturation, and insulin resistance; furthermore, it promotes the meiotic progression of germinal vesicle oocytes [11, 12]. Recent studies on PCOS patients showed a decrease of androgen levels and an improvement in ovulation and metabolic parameters after treatment with myo-ins and D-chiro-inositol (D-chiro-ins), which is another stereoisomeric form of inositol [13]. It was highlighted that very promising results were achieved administering myo-ins plus D-chiro-ins at their physiological range in plasma (i.e., 40 : 1) to ensure better clinical results in the PCOS therapy [13].

It is important to investigate the possibility of using AMH as a marker of some parameters: improved insulin resistance and decreased LH and androgen levels. This issue still needs further studies to reach a satisfactory framework [14].

Combined oral contraceptive (COC) pills, especially containing antiandrogen, are commonly used in the treatment of PCOS patients to suppress ovulation. In this study we focused our attention on the activity of myo-ins alone. Our primary outcome was to investigate the effect of myo-ins or COC on the clinical features, biochemical parameters, and AMH levels in PCOS patients. The secondary outcome of the study was to compare differences in changes after treatment with myo-ins or COC.

## 2. Methods

**Figure 1:**
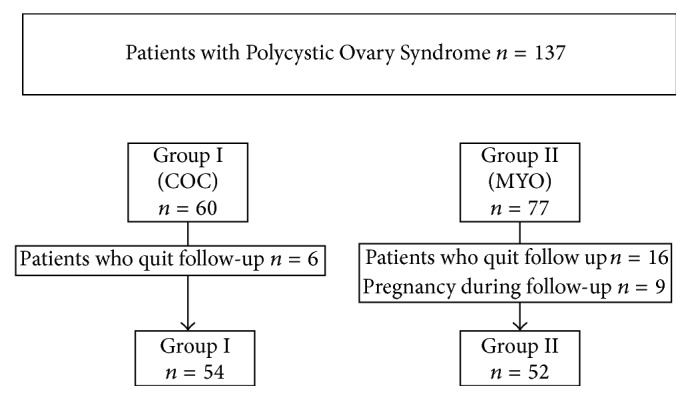
Flow chart of the study.

This is a randomized prospective trial carried out at the Department of Obstetrics and Gynecology (Dokuz Eylul University, Izmir, Turkey) between May 2013 and January 2014. This study started after the approval by the Clinical Research Ethics Committee of the Faculty of Medicine, Dokuz Eylul University, and the Turkish Health Ministry Drug and Medical Device Foundation. Informed written consent was obtained from all subjects. 137 patients diagnosed with Polycystic Ovary Syndrome and admitted to Dokuz Eylul University Faculty of Medicine, Department of Obstetrics and Gynecology, Division of Reproductive Endocrinology and Infertility Outpatient Clinic, were included in the study. The patients with odd numbers were allocated to COC group (*n* = 60), whereas the even ones to MYO group (*n* = 77) (Figure 1). The COC used was 2 mg cyproterone acetate and 0.035 mg ethinylestradiol (Diane 35; Schering AG, Istanbul, Turkey) daily. The COC was given for 21 days and in the following 7 days no drugs were given. This cycle was repeated for 3 months. In the myoinositol group, the product used contained 1 gram myoinositol and 100 *μ* gram folic acid (INOFOLIC; Lo.Li. Pharma, Rome, Italy). The drug was used twice a day continuously for 12–16 weeks. In initial assessment, for patients who had regular menstrual cycle for both MYO and COC group, the blood samples for hormonal assessment except progesterone were conducted on day 2 or 3 of the menstrual cycle. The progesterone levels were analyzed on the 21st day of the menstrual cycle. The assessment after three months was done when the treatment finished and the patients had her first menstrual cycle after three months of treatment. The blood samples were again taken at day 2 or 3 of the cycle. The progesterone levels were analyzed on the 21st day of the cycle. For both groups the patients were not under medication during the assessment of the hormone levels. In oligo/amenorrhoeic patients, blood samples were collected after withdrawal bleeding induced by oral progestin (5 mg medroxyprogesterone acetate twice a day; Tarlusal; Deva Holding A.Ş., Istanbul, Turkey).

The diagnosis of PCOS was made according to the Rotterdam criteria; as prescribed, two out of three features were detected in the patients: oligomenorrhea (fewer than six menstrual periods in the preceding year) and/or anovulation; clinical and/or biochemical signs of hyperandrogenism; presence of ≥12 follicles in each ovary measuring 2–9 mm in diameter; and/or increased ovarian volume (>10 mL) [15]. Smoking, hyperprolactinemia, hypogonadotropic hypogonadism, pregnancy, thyroid disease, congenital adrenal hyperplasia, androgen-secreting tumors, and Cushing's syndrome were ruled out during the screening phase. None among the enrolled patients had taken, at least in the previous six months, oral contraceptives, antiandrogens, or any drug that could influence carbohydrate metabolism. Clinical evidence of hyperandrogenism was determined by the Ferriman Gallwey score ≥8 that reveals the presence of hirsutism and/or acne. Biochemical hyperandrogenism was defined as androgen level increase.

Initial physical examination included weight, height, and waist and hip circumferences, to calculate waist/hip ratio (WHR) and body mass index (BMI). BMI calculated as kg/m^2^ was used as a measure of overall obesity. The WHR was used to assess the abdominal obesity. The waist circumference was measured at the midpoint of lowest margin of 12th rib and the lateral iliac crest during the normal expiration. The hip circumference was measured at the maximum distance between major trochanters. All anthropometric measurements were made by the same operator. Resting systolic and diastolic pressures were measured; after 2 minutes, the second measurement was performed and the mean values were determined.

Serum levels of fasting plasma glucose and insulin, C-reactive protein (CRP), anti-Mullerian hormone (AMH), apoprotein B (Apo B), apoprotein A1 (Apo A1), high density lipoprotein (HDL) and low density lipoprotein (LDL), total cholesterol, triglyceride, follicle stimulating hormone (FSH), luteinizing hormone (LH), total and free testosterone, dehydroepiandrosterone sulfate (DHEAS), and sex hormone binding globulin (SHBG) were measured. Normal insulin sensitivity was defined by fasting serum glucose and insulin levels with homeostatic model of insulin resistance (HOMA-IR). HOMA-IR was calculated by the formula: HOMA-IR = fasting blood glucose (mg/dL) × fasting insulin (*μ*IU/mL)/405.

Studies were performed within day 2 or 3 of the menstrual cycle. Fasting venous blood samples were taken between 08:00 am and 10:00 am after a 12-hour overnight fast. The blood samples were immediately centrifuged for 10 minutes and kept at −80°C in Eppendorf tubes until assayed.

Serum anti-Mullerian hormone (AMH) levels were analyzed with commercial kit according to manufacturer instructions based on the principle of competitive enzyme linked immunosorbent assay (ELISA) method (catalog number: CSB-E12756h, CUSABIO Biotech Co., USA). The microplate in the kit is precoated with goat-anti-rabbit antibody specific to AMH. Standard was reconstituted and prepared by serial dilution with sample diluent. Standards and undiluted samples are loaded into the appropriate microtiter plate wells with horseradish peroxidase (HRP) conjugated AMH and antibody specific to AMH. They were incubated for 60 minutes at 37°C. The competitive inhibition reaction was launched between HRP labeled AMH and unlabeled AMH with the antibody. Following a wash to remove any unbound substances, substrate A and B solutions were added and color develops in proportion to the amount of AMH. The color development is stopped and the intensity of the color is measured spectrophotometrically at a wavelength of 450 nm. A standard curve of known concentration (0, 0.375, 1.31, 4.69, 28.12, and 150 ng/mL) of AMH was established and the concentration of analyte in the samples was calculated accordingly. The ELISA assays of AMH had a sensitivity of 0.375 ng/mL; a detection range of 0.375–150 ng/mL; intra-assay coefficient of variation <%10, interassay coefficient of variation <%15, respectively.

Ovarian volume measurement was made by Medison Sono Ace X6 ultrasound system. For transvaginal ultrasonography, the probes used were EV4–9/10ED center frequency 6.5 mHz and ER4–9/10ED center frequency 6.5 mHz. For virgin patients transabdominal ultrasonography was used. The probe of the transabdominal ultrasonography was C2–8, center frequency 5 mHz. The presence of ≥12 follicles in each ovary measuring 2–9 mm in diameter was recorded. The total number of these follicles was accepted as follicle count. Longitudinal, transverse, and anteroposterior ovarian diameters were measured and multiplied by 0.5 to calculate the ovary volume.

Data were analyzed by using Statistical Program for Social Sciences (SPSS, version 16). The level of significance was accepted when *p* < 0.05. In Table 1, the test used was independent *t*-test. In Table 2, the test used was independent *t*-test. In Table 3, the test used was paired samples test. In Table 4, the test used was paired samples test.

## 3. Results

106 patients were analyzed, among them 54 patients were COC receivers (group 1), and 52 were myo-ins receivers (group 2). Baseline demographic, clinical characteristics, and ultrasound results of all patients are presented in Table 1. The mean age was 22.79 ± 4.13 years and 24.44 ± 4.78 years for groups 1 and 2, respectively. In group 1, 41 (75.9%) of 54 patients showed menstrual irregularity. At the end of the study period, patients in group 1 showed no menstrual irregularity, whereas the decrease of menstrual irregularity in group 2 was from 40 (76.9%) to 8 (15.4%) patients.

The baseline biochemical and hormonal values showed that patient groups were matched in these parameters except sex hormone binding globulin (SHBG) level which was higher in group 1 (Table 2).

Increase in HDL and decrease in LH and LH/FSH ratio were determined statistically significant only in group 1 when compared with the baseline values (for all *p* > 0.05) (Table 3). In group 2, the reduction of fasting glucose, LDL, DHEAS, total cholesterol, and prolactin levels was statistically significant (for all *p* < 0.05) (Table 3). Other parameters, such as total testosterone, DHEAS, and fasting glucose, were statistically improved in group 2, while no particular improvement was present in group 1 (Table 3). After 3-month treatment, AMH (for group 1 *p* < 0.001; for group 2 *p* = 0.002) levels as well as ovarian volumes, ovarian antral follicle count, and total antral follicle counts showed a statistically significant decrease in both groups (for all *p* < 0.001) (Table 3). When we evaluate progesterone levels at the end of the treatment, group 1 showed a statistically significant decrease whereas group 2 showed a statistically significant increase (for group 1 *p* = 0.014; for group 2 *p* < 0.001).

The lessening of ovarian volumes and AMH levels was statistically significantly more in the MYO group than COC group (for AMH; *p* = 0.048, for ovarian volumes *p* = 0.040) (Table 4).

## 4. Discussion

There are some therapies for Polycystic Ovary Syndrome, acting in different metabolic pathways. This prospective study observed the change in clinical, biochemical parameters and anti-Mullerian hormone levels after treatment with two different drug supplementations, myoinositol and combined oral contraceptives. Study data demonstrated that myo-ins regimen in PCOS patients positively affects metabolic parameters and modulates various hormonal factors deeply involved in the reproductive function and ovulation such as anti-Mullerian hormone.

It is well known that PCOS is characterized by hyperandrogenism and irregular menstrual cycles. Concerning menstrual irregularity, using myo-ins Papaleo et al. [16] and Gerli et al. [17] observed an improvement of 88% (after six months) and 70% (after 14 weeks). In the current study, 76.9% of patients in group 2, treated with myo-ins, had menstrual irregularity initially. Use of myo-ins was associated with a significant improvement, and menstrual irregularity was strongly reduced in the study population. This effect may be explained by the specific protein phosphorylation processes via protein kinase C, which modulates various cellular processes as a second messenger system.

Anti-Mullerian hormone is secreted primarily in the small antral follicles and AMH measurements correspond to granulosa cell activity, total antral follicle count, and ovarian volume. AMH has been also reported to be increased in PCOS women [18]. In addition, several studies showed that AMH might correlate with the severity of this syndrome [19]. In recent researches, positive correlations between AMH levels, ovarian volume, and total antral follicle count were demonstrated [20–22]. Several studies in the literature support that COC treatment decreases ovarian volume and antral follicle [23, 24]. Our analysis showed significant reduction in total antral follicle count and ovarian volume after treatment with COC and myo-ins (for both groups *p* < 0.001). In line with our study, the two researches of Genazzani demonstrated reduction in ovarian volume after myo-ins administration. However, they reported no changes in the total antral follicle count after myo-ins treatment [25, 26]. The predictability of a successful therapy can be monitorized by changes in AMH levels. Although there are no previously published data about changes in serum AMH levels during myo-ins therapy, there are several studies that observed AMH changes after treatment with COC [27–29]. Li et al. examined 95 women in five different groups and they found no significant change in AMH levels before and after treatment with combined oral contraceptives [27]. In contrast, decrease in AMH levels after COC was shown in previous studies and it has been confirmed in our study [28, 29]. The decline in AMH levels may be related to the suppression of ovarian function with COC. Research has been mainly focused on COC's activity and there are no clinical studies on myo-ins effect on AMH levels. Genazzani et al. reported that myo-ins supplementation, in PCOS patients, affected metabolic parameters such as insulin sensitivity and modulated positively hormonal factors like LH, FSH, and testosterone [25]. There was one retrospective study which showed decreased AMH levels after D-chiro-ins [30]. To our knowledge, the present study is the first one in the scientific literature indicating statistically significant decrease in AMH levels after 12–16 weeks of myo-ins use. We believe that suppression in AMH levels may be explained by the reduction of total antral follicle count and ovarian volume. However, the mechanism between decrease in total antral follicle count and myo-ins therapy is not clearly understood yet. We hypothesize that whereas hyperinsulinemia may stimulate the development of antral follicles and recover the sensitivity of granulosa cells to FSH, therefore leading to increase in the number of follicles, ovarian volume, and AMH levels, myo-ins may improve clinical and hormonal features of PCOS patients by enhancing insulin sensitivity that decreases hyperinsulinemia.

In our trial, we compared the ovarian volumes, total antral follicle count, and serum AMH levels after treatment with COC and myo-ins. Patients in the study group showed significantly more relevant reduction in AMH levels (*p* = 0.048) and ovarian volume (*p* = 0.040) than the COC group. The decrease in total antral follicle count for myo-ins and COC group was similar (*p* = 0.356). Based on our data, we believe that myo-ins is more effective compared to COC in lowering AMH levels and ovarian volumes. Additionally myo-ins has much less side effects and is without contraindications. Myoinositol cannot replace COC for contraception and may be preferred for patients with infertility who desire to conceive pregnancy. Also, myoinositol can be an option in patients with hirsutism, oligoanovulation, and symptoms of hyperandrogenism who have a contraindication to use COC. Further studies are needed to compare myoinositol and antiandrogenic drugs. Myoinositol is not a primary treatment; it is an adjunct therapy to improve insulin resistance.

In the present study, we also observed the effect of COC and myo-ins on fasting insulin, fasting glucose, and HOMA-IR. The results showed no significant change in insulin or HOMA-IR in both groups. Only PCOS patients, who received myo-ins, presented a significant reduction in glucose levels. However, we know that our study had a too short duration to be capable of detecting myo-ins effect on insulin and HOMA-IR. In contrast with our data, Zacchè et al. demonstrated that fasting insulin and HOMA-IR values were decreased after myo-ins treatment [31]. Villaseca et al. reported that COC did not affect fasting insulin or fasting glucose levels or HOMA-IR [32]. In Minozzi et al.'s study, patients were divided into two groups as COC + myo-ins receivers and only COC receivers. The results showed a significant reduction in glucose levels only in COC + myo-ins group, whereas no significant change was observed in COC group. The results of Minozzi et al.'s prospective study showed that the combination of COC and myo-ins was a more effective treatment for clinical symptoms of PCOS and in controlling endocrine disorders and insulin resistance [33].

Many studies have been carried out in PCOS patients with high serum androgen levels to determine the clinical implications. Most of the agents used in the treatment aimed to reduce serum androgen levels. The results of this study showed that myoinositol treatment was able to improve some key parameters such as the total testosterone level. The reduction of testosterone is a key outcome in the management of PCOS women, because of the typical symptoms related to the hyperandrogenic status, affecting the PCOS pathway. These results could be due to the insulin sensitizing action of myoinositol and the sequent downregulation of the androgens production at ovarian level [31]. The distinct effect of myoinositol on testosterone, AMH, and ovarian volume is surprising and this could be explained by a reduction in hyperinsulinism. In our study, we did not observe significant reduction in insulin and HOMA-IR, but still there is a decrease in HOMA-IR blood levels. To observe the significant change, the study period should be extended. Also, the number of the patients in the study should be raised.

Zacchè et al. demonstrated a decrease in free testosterone and total testosterone after myo-ins treatment, but no significant change in androstenedione levels [31]. When we evaluate the changes in androgen levels in our study, we have seen that myo-ins treatment reduced DHEA-S and total testosterone levels, but no significant differences in SHBG and androstenedione were found. In the present study, whereas COC group showed a significant increase in SHBG levels, statistical significance was not observed in the increase of DHEAS, free testosterone, and androstenedione and in the reduction in total testosterone levels. Literature data indicate that if the treatment duration was extended, changes in androgen levels might become significant. Therefore, it is more appropriate planning a long term therapy in the management of androgen excess, anovulation, and insulin resistance.

## 5. Conclusion

This study shows that the combination of myoinositol plus folic acid should be considered in the treatment of PCOS patients. Considering previous studies, myo-ins has also reduced hirsutism, yet more slowly than COC. Studies in the literature indicate that myoinositol exerts positive effects on insulin resistance, but further researchers are still required to clarify the mechanism. The present study is the first one reporting that myoinositol is superior to COC in terms of lowering androgens levels. Also, myoinositol effect on ovarian volume and AMH levels is remarkable when compared to COC.

## Figures and Tables

**Table 1:** Comparison of demographic and clinical characteristics of group 1 and group 2.

	GROUP 1-COC (*n* = 54)	GROUP 2-MYO (*n* = 52)	^*∗*^ *p*
Age (years)	22.79 ± 4.13	24.44 ± 4.78	0.061
Gravida	0.31 ± 0.88	0.30 ± 0.64	0.962
Parity	0.16 ± 0.46	0.11 ± 0.37	0.536
BMI (kg/m^2^)	23.79 ± 4.24	25.33 ± 5.20	0.098
WHR	0.76 ± 0.15	0.83 ± 0.22	0.064
Mean artery pressure (mmHg)	82.16 ± 12.82	87.50 ± 10.31	**0.020**
Ferriman Gallwey score	12.53 ± 5.57	14.38 ± 6.41	0.116
Right ovary volume (cm^3^)	9.50 ± 2.92	9.84 ± 2.75	0.545
Left ovary volume (cm^3^)	8.93 ± 3.27	9.32 ± 2.42	0.495
Total ovary volume (cm^3^)	18.44 ± 5.89	19.16 ± 4.58	0.486
Right ovary antral follicle count	16.09 ± 5.62	16.96 ± 6.38	0.458
Left ovary antral follicle count	14.81 ± 5.64	15.65 ± 5.71	0.449
Total antral follicle count	30.90 ± 10.27	32.61 ± 10.50	0.399

COC: combined oral contraceptives, MYO: myoinositol + folic acid, BMI: body mass index, and WHR: waist hip ratio. Independent *t*-test.

**Table 2:** Comparison of baseline biochemical and hormonal parameters of patient groups.

	GROUP 1-COC (*n* = 54)	GROUP 2-MYO (*n* = 52)	^*∗*^ *p*
Fasting glucose (mg/dL)	85.42 ± 8.60	86.48 ± 8.85	0.535
Fasting insulin (*μ*IU/dL)	11.14 ± 8.87	12.06 ± 10.40	0.626
HOMA-IR	2.42 ± 2.08	2.62 ± 2.30	0.649
HDL (mg/dL)	51.14 ± 11.99	50.32 ± 12.50	0.731
LDL (mg/dL)	103.48 ± 31.53	112.84 ± 34.76	0.149
TG (mg/dL)	118.53 ± 67.18	119.44 ± 60.86	0.942
Total cholesterol (mg/dL)	180.51 ± 38.25	183.92 ± 35.55	0.636
CRP (mg/L)	2.54 ± 3.04	3.02 ± 3.27	0.437
Apo B/A1	0.62 ± 0.23	0.65 ± 0.24	0.537
DHEAS (*μ*g/dL)	303.97 ± 138.57	318.29 ± 178.02	0.644
C-peptide (ng/mL)	2.84 ± 1.71	2.61 ± 1.41	0.461
Total testosterone (ng/dL)	1.10 ± 3.72	0.80 ± 0.47	0.573
Free testosterone (pg/mL)	2.01 ± 1.04	2.39 ± 1.72	0.176
Androstenedione (ng/mL)	4.25 ± 2.97	4.82 ± 3.79	0.397
17-OH progesterone (ng/mL)	1.22 ± 0.65	1.03 ± 0.58	0.119
SHBG (nmol/L)	59.40 ± 47.65	39.14 ± 29.54	**0.010**
Progesterone (ng/mL)	1.27 ± 1.88	1.37 ± 2.88	0.824
Estradiol (pg/mL)	63.98 ± 58.16	49.41 ± 26.42	0.102

COC: combined oral contraceptives; MYO: myoinositol + folic acid; HOMA-IR: Homeostatic Model Assessment-Insulin Resistance; HDL: high density lipoprotein; LDL: low density lipoprotein; TG: triglyceride; CRP: C-reactive protein; Apo B/A1: apoprotein B/A1; DHEAS: dehydroepiandrosterone sulfate; SHBG: sex hormone binding globulin; FSH: follicular stimulating hormone; LH: luteinizing hormone; TSH: thyroid stimulating hormone; AMH: anti-Mullerian hormone. Independent *t*-test.

**Table 3:** Comparison of changes in all parameters after treatment between the groups.

	GROUP 1-COC (*n* = 54)			GROUP 2-MYO (*n* = 52)		
	Month 0	Month 3	^*∗*^ *p*	Month 0	Month 3	^*∗*^ *p*
BMI (kg/m^2^)	23.79 ± 4.24	23.95 ± 4.28	0.166	25.33 ± 5.20	25.23 ± 5.02	0.656
WHR	0.76 ± 0.15	0.77 ± 0.16	0.175	0.83 ± 0.22	0.82 ± 0.21	0.230
Ferriman Gallwey score	12.53 ± 5.57	12.09 ± 5.40	**<0.001**	14.38 ± 6.41	14.40 ± 6.31	0.859
Fasting glucose (mg/dL)	85.42 ± 8.60	85.40 ± 9.25	0.990	86.48 ± 8.85	82.55 ± 14.03	**0.028**
Fasting insulin (*μ*IU/dL)	11.14 ± 8.87	10.57 ± 8.81	0.521	12.06 ± 10.40	10.93 ± 12.57	0.531
HOMA-IR	2.42 ± 2.08	2.33 ± 2.09	0.633	2.62 ± 2.30	2.36 ± 2.97	0.527
HDL (mg/dL)	51.14 ± 11.99	54.50 ± 12.15	**0.016**	50.32 ± 12.50	50.13 ± 10.88	0.870
LDL (mg/dL)	103.48 ± 31.53	99.12 ± 27.51	0.102	112.84 ± 34.76	105.55 ± 29.15	**0.031**
TG (mg/dL)	118.53 ± 67.18	123.20 ± 60.87	0.554	119.44 ± 60.86	117.00 ± 69.45	0.703
Total cholesterol (mg/dL)	180.51 ± 38.25	179.74 ± 34.01	0.822	183.92 ± 35.55	179.63 ± 28.18	0.200
DHEAS (*μ*g/dL)	303.97 ± 138.57	295.22 ± 116.51	0.492	318.29 ± 178.02	284.16 ± 136.73	**0.043**
C-peptide (ng/mL)	2.84 ± 1.71	2.75 ± 2.05	0.770	2.61 ± 1.41	2.36 ± 1.46	0.220
Total testosterone (ng/dL)	1.10 ± 3.72	1.30 ± 5.49	0.826	0.80 ± 0.47	0.54 ± 0.22	**<0.001**
Free testosterone (pg/mL)	2.01 ± 1.04	1.82 ± 0.83	0.153	2.39 ± 1.72	2.22 ± 0.84	0.460
Androstenedione (ng/mL)	4.25 ± 2.97	3.74 ± 1.95	0.098	4.82 ± 3.79	4.75 ± 3.10	0.870
SHBG (nmol/L)	59.40 ± 47.65	74.16 ± 53.78	**0.030**	39.14 ± 29.54	42.22 ± 25.44	0.479
Progesterone (ng/mL)	1.27 ± 1.88	0.70 ± 0.59	**0.014**	1.37 ± 2.88	4.41 ± 4.35	**<0.001**
Estradiol (pg/mL)	63.98 ± 58.16	46.59 ± 32.20	0.051	49.41 ± 26.42	47.03 ± 25.04	0.635
FSH (mIU/mL)	5.20 ± 2.04	5.59 ± 1.77	0.246	5.46 ± 3.78	5.89 ± 1.61	0.397
LH (mIU/mL)	7.61 ± 4.19	4.43 ± 2.85	**<0.001**	8.12 ± 5.17	8.52 ± 6.57	0.680
LH/FSH ratio	1.51 ± 0.70	0.81 ± 0.47	**<0.001**	1.66 ± 0.94	1.45 ± 0.87	0.146
Prolactin (ng/mL)	12.88 ± 7.19	13.66 ± 4.67	0.370	13.33 ± 6.04	10.81 ± 5.00	**0.001**
TSH (*μ*U/mL)	1.70 ± 0.83	1.79 ± 0.82	0.481	1.65 ± 0.71	1.83 ± 0.77	0.124
AMH (ng/mL)	9.39 ± 6.60	8.51 ± 6.20	**<0.001**	11.51 ± 11.50	9.07 ± 9.32	**0.002**
Right ovary volume (cm^3^)	9.50 ± 2.92	8.12 ± 2.70	**<0.001**	9.84 ± 2.75	7.81 ± 2.43	**<0.001**
Left ovary volume (cm^3^)	8.93 ± 3.27	7.94 ± 3.03	**<0.001**	9.32 ± 2.42	7.38 ± 1.69	**<0.001**
Total ovary volume (cm^3^)	18.44 ± 5.89	16.06 ± 5.51	**<0.001**	19.16 ± 4.58	15.19 ± 3.56	**<0.001**
Right ovary follicle count	16.09 ± 5.62	11.05 ± 5.65	**<0.001**	12.96 ± 6.38	10.82 ± 6.02	**<0.001**
Left ovary follicle count	14.81 ± 5.64	10.75 ± 5.97	**<0.001**	15.65 ± 5.71	11.05 ± 5.95	**<0.001**
Total antral follicle count	30.94 ± 10.27	21.81 ± 10.99	**<0.001**	32.61 ± 10.50	21.88 ± 11.23	**<0.001**

COC: combined oral contraceptives; MYO: myoinositol + folic acid; BMI: body mass index; WHR: waist hip ratio; HOMA-IR: Homeostatic Model Assessment-Insulin Resistance; HDL: high density lipoprotein; LDL: low density lipoprotein; TG: triglyceride; DHEAS: dehydroepiandrosterone sulfate; SHBG: sex hormone binding globulin; FSH: follicular stimulating hormone; LH: luteinizing hormone; TSH: thyroid stimulating hormone; AMH: anti-Mullerian hormone. Paired samples test.

**Table 4:** Change in AMH levels, ovary volumes, and total antral follicle counts between group 1 and group 2.

	Δ_COC_ (*n* = 54)	Δ_MYO_ (*n* = 52)	^*∗*^ *p*
AMH (ng/mL)	0.88 ± 1.72	2.44 ± 5.45	**0.048**
Total ovary volume (cm^3^)	2.37 ± 2.81	3.97 ± 4.84	**0.040**
Total antral follicle count	9.09 ± 6.77	10.73 ± 10.98	0.356

COC: combined oral contraceptives: MYO: myoinositol + folic acid; AMH: anti-Mullerian hormone. Δ indicates change. Paired samples test.
