# Corrigendum to “Different Effects of Myoinositol plus Folic Acid versus Combined Oral Treatment on Androgen Levels in PCOS Women”

**DOI:** 10.1155/2018/7502102

**Published:** 2018-09-10

**Authors:** Ali Cenk Ozay, Ozlen Emekci Ozay, Recep Emre Okyay, Erkan Cagliyan, Tuncay Kume, Bulent Gulekli

**Affiliations:** ^1^Aksehir State Hospital, Konya, Turkey; ^2^Department of Obstetrics & Gynecology, Medical School, Dokuz Eylul University, Izmir, Turkey; ^3^Department of Biochemistry, Medical School, Dokuz Eylul University, Izmir, Turkey

In the article titled “Different Effects of Myoinositol plus Folic Acid versus Combined Oral Treatment on Androgen Levels in PCOS Women” [1], “Competing Interests” should read as follows:

Publication was sponsored by ITF Ilac. The company was not involved in the study or the preparation of the article.

## References

[1] Ozay A. C., Emekci Ozay O., Okyay R. E., Cagliyan E., Kume T., Gulekli B. (2016). Different effects of myoinositol plus folic acid versus combined oral treatment on androgen levels in PCOS women. *International Journal of Endocrinology*.

